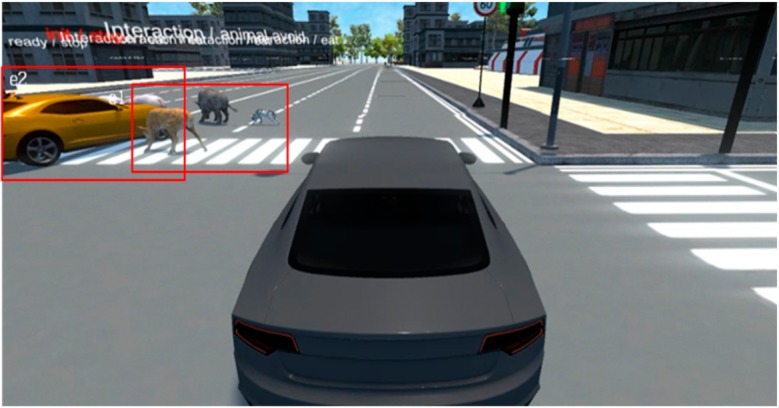# Multiple Event-Based Simulation Scenario Generation Approach for Autonomous Vehicle Smart Sensors and Devices

**DOI:** 10.3390/s19204456

**Published:** 2019-10-14

**Authors:** Jisun Park, Mingyun Wen, Yunsick Sung, Kyungeun Cho

**Affiliations:** Department of Multimedia Engineering, Dongguk University-Seoul, Seoul 04620, Korea; jisun@dongguk.edu (J.P.); wmy_dongguk@dongguk.edu (M.W.); sung@mme.dongguk.edu (Y.S.)

**Keywords:** scenario generation, autonomous vehicle, smart sensor and device, deep learning

## Abstract

Nowadays, deep learning methods based on a virtual environment are widely applied to research and technology development for autonomous vehicle’s smart sensors and devices. Learning various driving environments in advance is important to handle unexpected situations that can exist in the real world and to continue driving without accident. For training smart sensors and devices of an autonomous vehicle well, a virtual simulator should create scenarios of various possible real-world situations. To create reality-based scenarios, data on the real environment must be collected from a real driving vehicle or a scenario analysis process conducted by experts. However, these two approaches increase the period and the cost of scenario generation as more scenarios are created. This paper proposes a scenario generation method based on deep learning to create scenarios automatically for training autonomous vehicle smart sensors and devices. To generate various scenarios, the proposed method extracts multiple events from a video which is taken on a real road by using deep learning and generates the multiple event in a virtual simulator. First, Faster-region based convolution neural network (Faster-RCNN) extracts bounding boxes of each object in a driving video. Second, the high-level event bounding boxes are calculated. Third, long-term recurrent convolution networks (LRCN) classify each type of extracted event. Finally, all multiple event classification results are combined into one scenario. The generated scenarios can be used in an autonomous driving simulator to teach multiple events that occur during real-world driving. To verify the performance of the proposed scenario generation method, experiments using real driving video data and a virtual simulator were conducted. The results for deep learning model show an accuracy of 95.6%; furthermore, multiple high-level events were extracted, and various scenarios were generated in a virtual simulator for smart sensors and devices of an autonomous vehicle.

## 1. Introduction

Recently, autonomous vehicles have been a big trend in the development of advanced countries worldwide [1,2,3]. Especially, studies on the perception system of an autonomous vehicle using smart sensors and devices are being active widely because perception is one of key element of autonomous vehicles. Recently in the autonomous vehicle industry, smart sensors and devices of autonomous vehicles have been trained via virtual self-driving simulators that apply the deep learning technique to reduce development costs and time and secure safety [4,5,6,7,8,9,10]. The virtual autonomous driving simulators provide color image (RGB), depth, Lidar, and radar data to train autonomous vehicle’s smart devices and sensors [4,5]. To enable an autonomous vehicle to run in real environments, it is critical to train a self-driving car for a variety of driving environments in advance. Furthermore, it is also essential to learn scenarios reflecting a wide range of situations that may occur in the real world. As an example, when an autonomous vehicle runs on a road in an urban area, the car needs to be trained for scenarios with several people walking on the streets. When an autonomous vehicle runs on an expressway, the car must be trained for scenarios of diverse types of situations that can occur by interaction among cars on the expressway. 

Existing studies are based on the scenario generation approaches for autonomous vehicle generated scenarios based on real driving data acquired from the real environment or by using self-driving scenario generation modeling based on expert knowledge [11,12,13,14,15,16]. However, such approaches require a high ratio of manual processing, which increases the development costs and time for the self-driving simulator. Thus, it is beneficial to investigate the approach for generating the scenarios by using deep learning video analysis for automatically generating a wide range of realistic driving scenarios through the collection and analysis of real driving data without scenario generation modelling. 

The deep learning approach for analyzing driving data is limited as it can only analyze the actions of one object [17,18]. As an example, when two individuals are talking and walking, and extraction is to be performed based on a single object, only two walking individuals can be extracted. Such an approach cannot analyze advanced events, including multiple objects and interaction.

This paper proposes an approach to generate the training scenario for autonomous vehicle smart sensors and devices including multiple events while considering multiple objects based on the automatic analysis of a driving video by using two types of deep learning approaches. An event comprises the list of objects included in one specific situation and the actions of each object.

The first step is to extract the areas of objects existing in a driving video input to Faster-region based convolution neural network (Faster-RCNN) [19]. Faster-RCNN is real-time object detection network. Next, the high-level event area is estimated while considering the extracted areas of objects. Then, the events are analyzed using long-term recurrent convolution networks (LRCN) [20] based on the high-level event areas extracted. LRCN classifies the video class by convolutional neural network (CNN) and long short-term memory (LSTM). Finally, the analyzed events are integrated into one scenario. The generated scenario is delivered to the virtual simulator for the learning of an autonomous vehicle, and the relevant scenario is deployed in front of an autonomous vehicle. 

This paper contributes to future research as follows. First, a scenario was successfully generated via automatic analysis using deep learning for training and testing of autonomous vehicle’s smart sensors and devices. Next, the approach enables the sophisticated analysis of events including interactions among multiple objects as well as the analysis of only a single action by each object. Finally, it is possible to generate higher-level scenarios including multiple events. 

Section 2 in this paper describes the existing research on scenario generation for an autonomous vehicle and the video analysis approach based on deep learning. Section 3 discusses the scenario generation approach proposed in this paper, which extracts high-level events using deep learning-based video analysis. Section 4 describes the experiments on the proposed approach and the results, and Section 5 presents the conclusion and directions for further study.

## 2. Related Works

This section summarizes the existing studies on driving scenario generation approaches and deep learning-based driving video analysis approaches. Then, the necessity for the approach proposed herein is explained. 

### 2.1. Driving Scenario Generation Approach

Several driving simulators has been investigated for development and verification of an autonomous vehicle. The field of driving scenario generation for the operation of autonomous vehicles has recently drawn substantial attention [11,12,13,14,15,16]. Research on driving scenario generation is largely classified into model-based and data-based scenario generation. 

The model-based scenario generation approach defines driving elements, including traffic lane, car, pedestrian, and accident events, in advance as well as scenarios depending on those elements. In [11] the authors plan movements and generate scenarios by using the action tree of each car based on the accident scenario defined in scripts, and the research in [12] predefines the accident scenario between a car and a pedestrian in the intersection and generates the scenarios. In [13] the authors generate the scenarios based on an analysis of real car accidents and survey data from ‘NMVCCS’ and in [14] the authors implement the ontology on the driving environment and generate scenarios based on that ontology. For the model-based scenario generation approach above, a more complicated scenario requires higher scenario modeling time and cost. Moreover, it is very difficult to modify or supplement a scenario after it is generated using the approach above. 

The data-based scenario generation approach generates a scenario only from real driving data. The research presented in [15] generates the scenarios by using data recorded by experts after analyzing information on a lane type, car, and pedestrian based on a driving video recorded for 30 h on a real road. In [16] the authors acquire real driving data by using laser sensors and cameras and apply the data to the virtual environment simulator. The scenario generation based on real driving data as explained above enables an autonomous vehicle to learn practical scenarios but has disadvantages related to the required time and cost of obtaining real driving data

For model-based and data-based driving scenario generation approaches as described above, it is inevitable that the more diverse types of scenarios that are generated, the greater the required time and cost. Accordingly, this paper attempts to address the disadvantages of existing studies by developing an approach to automatically analyze real driving data and generate diverse types of scenarios, including multiple events using analysis results.

### 2.2. Deep Learning-Based Driving Video Analysis Approach

The studies analyzing videos by using deep learning have been conducted actively [17,18], and in particular, the dataset for training an autonomous vehicle has been continuously increasing [19,20]. Most studies analyzing deep learning-based videos extracted a specific vector from a series of video frames by using a CNN and integrated the extracted specific vectors around the time axis. However, most studies extracted each object and analyzed only the actions of that object. Furthermore, only one event was analyzed per video. The research presented in [17] extracted RGB image-specific vectors and optical flow vectors per frame by using CNN, entered that extracted specific vectors into CNN to fuse two vectors, and classified it into one event class by using support vector machine (SVM). In [18] the authors extracted specific vectors from RGM images and optical flow images per frame in the video and segmented trajectory data by using CNN. Subsequently, it integrated and estimated three specific vectors and classified the result as one event by SVM. 

As described above, the existing studies on deep learning-based driving video analysis analyzed the actions of only one object, rather than advanced events including interaction. They could analyze only a single event per video. This paper proposes an approach to extract and analyze multiple events that are more advanced.

## 3. Multi-Event-Based Scenario Generation Approach

**Figure 1:**
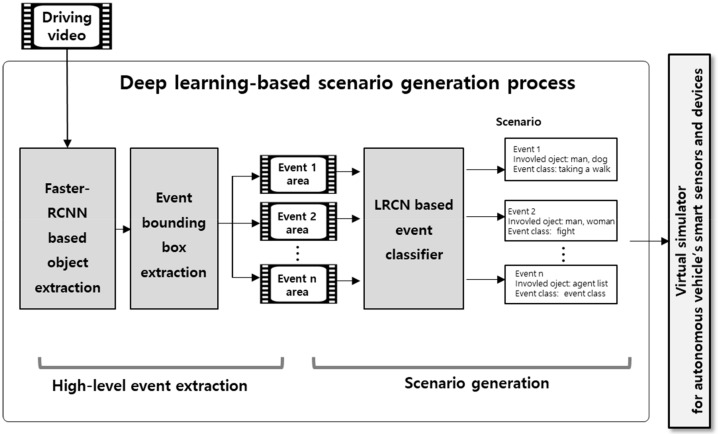
Proposed multi-event-based scenario generation approach.

This paper proposes an approach to generate scenarios for training autonomous vehicle’s smart sensors and devices by extracting and analyzing multiple events from driving video using deep learning methods. Figure 1 illustrates the scenario generation process based on the deep learning video analysis approach proposed in this paper. The first step is to extract a high-level event area to detect the objects existing in a video by using Faster-RCNN, which is optimal for detecting objects with the first frame of the input. The objects whose bounding boxes overlap among detected objects are extracted as one event area. Next, the scenario generation step analyzes the images extracted based on the event area in the previous step by using LRCN, which is a type of deep learning-based video classification model, and generates the scenarios for self-driving learning based on the analysis. The generated scenario is finally used as the input data for the self-driving simulator. In the proposed approach, the events are presented as the list of objects and high-level event class included in the relevant events, and scenarios are presented as the list of events. Figure 1 shows the entire process of the proposed approach.

### 3.1. High-Level Event Area Extraction Step

**Figure 2:**
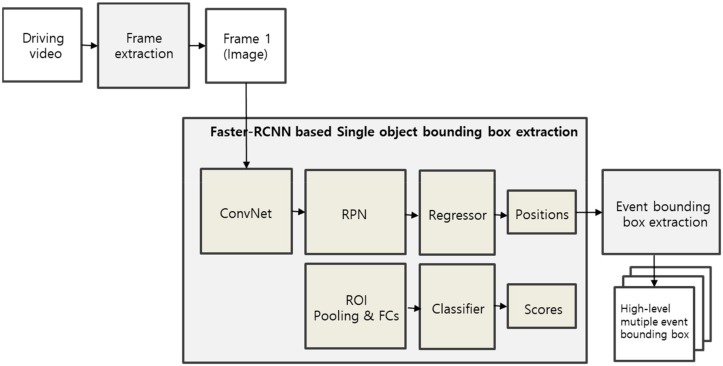
Multiple high-level events extraction process.

Figure 2 shows the process to extract more optimum event areas in the driving data. The process comprises the object detection and event area integration in that sequence. For the object detection task, the first image (frame) is received using the Faster-RCNN approach, and the areas of dynamic objects such as a person, car, and animal, which can be the subject of an event, are extracted. The Faster-RCNN includes Convolutional neural network (ConvNet), Region proposal network (RPN), Region of Interest pooling, regression and classification layer. After extracting an event bounding box based on a single object area, it is difficult to extract high-level events including interactions between objects. The proposed approach enables the extraction of higher-level event areas by integrating neighboring single object bounding boxes into one even bounding box. 

**Figure 3:**
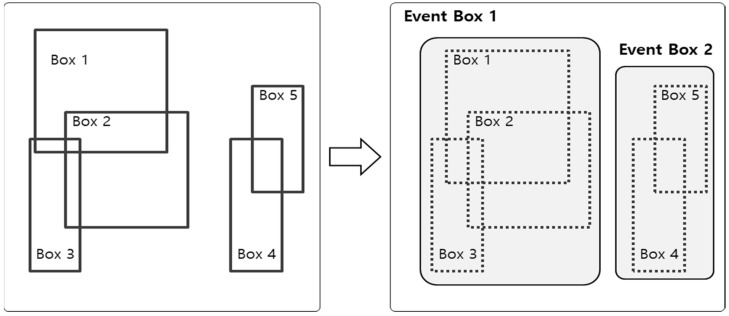
High-level event area extraction process.

Figure 3 illustrates the approach to integrate the object areas detected using Faster-RCNN into the high-level event area. The first step sorts the boxes whose areas are overlapped among bounding boxes of detected objects. Next, the top and left sides of an event bounding box are set to the minimum value among the bounding boxes of overlapped objects, and the right and bottom sides are set to the maximum value among the bounding boxes of overlapped objects. Algorithm 1 is the algorithm to integrate event areas. The overlapped bounding boxes of objects are integrated into one event bounding box through Algorithm 1; subsequently, multiple event areas are extracted based on the integration results.

**Algorithm 1.** Event Area Integration Algorithm***E***: ***An event includes the list of objects included in the event and the class of the event******O***: ***Objects including persons, animals, or cars***Initialize ***E***
GET ***O*** For each i in ***O***
  IF ***E*** = [] THEN increment new e   For each j in ***E***
   IF $O_{i}$ overlaps $E_{j}$ THEN    IF $O_{i}$ > $E_{j}$ THEN      merge $O_{j}$ into $E_{j}$
  ELSE increment new e  ENDFOR ENDFOR

### 3.2. Scenario Generation Step

**Figure 4:**
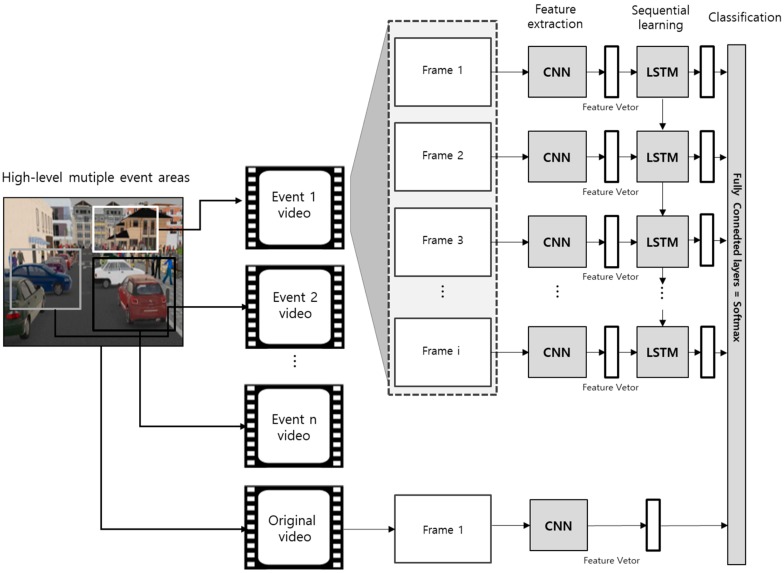
Long-term recurrent convolution networks (LRCN)-based event classification model structure.

The scenario generation process based on multiple event images extracted comprises the LRCN-based event classification task and the scenario generation task depending on the classification results. As shown in Figure 4, the deep learning model structure classifying events based on LRCN comprises the combination of CNN extracting the features of the extracted images and LSTM learning the sequential data. The specific feature vectors per frame are extracted via CNN after receiving individual frames of each event image based on the extracted event areas. Next, the result values acquired after entering the specific vectors per frame to LSTM in consecutive order, which are classified into the event label via the Fully Connected Layer. As the event areas include only a part of the full image, the specific vectors in the first frame of the original video on the full area as well as the feature value of event area frame are entered into the last Fully Connected Layer to include the features of full images, including weather and road type.

Multiple event images are classified by repeating the process above and stored as one scenario. A scenario is the list of events, and each event includes the types of objects contained in the relevant event and the high-level event class of the relevant event. A list of scenario elements is presented in Table 1.

**Figure 5:**
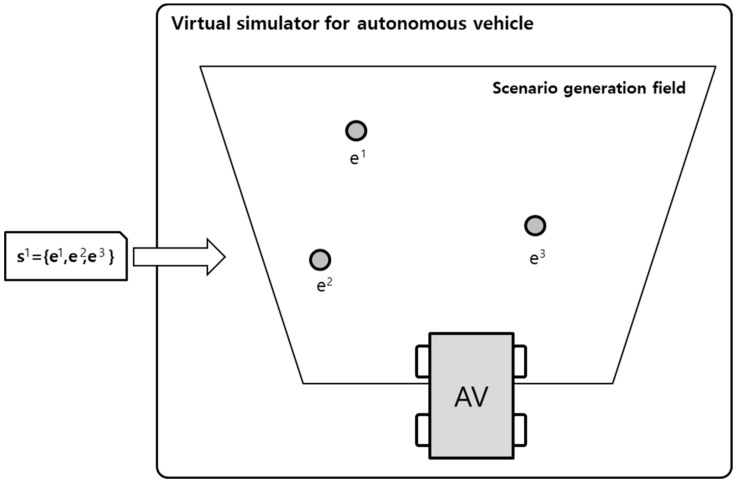
Execution of multiple events in a virtual simulator through the scenario input.

After a scenario is generated in the structure described above, the relevant data is transferred to the virtual simulator, as illustrated in Figure 5. The input scenarios execute the events in front of an autonomous vehicle depending on the object list and action contained in each event. The virtual simulator operates the input scenario and then the autonomous vehicle learn the scenario by training their virtual sensing device data such as RGB-D, Lidar, and Radar data.

## 4. Experiments and Analysis

This section describes the experiments and analysis of the scenario generation approach based on the deep learning image analysis proposed herein to verify its performance. To this end, the experimental environment is described and learning data is presented. The results of the algorithm extracting multiple event areas are compared to those of the existing Faster-RCNN. Next, the image analysis algorithm performance proposed herein is compared to that of the existing RCNN algorithm and analyzed. Finally, the final extracted scenario was executed in the simulator, which was constructed for the experiment, and the results are analyzed.

### 4.1. Experiment Environment and Training Data

**Figure 6:**
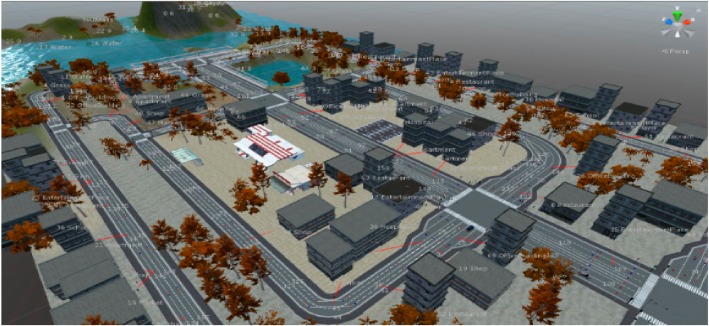
Virtual simulator environment for an autonomous vehicle to train.

The proposed method’s development environment was implemented on a computer with Intel i5, Nvidia GTX 1070 GPU, and DDR 5 H/W. The scenario generation model utilizing the deep learning-based video analysis was implemented in Keras (Backend-Tensorflow), which is a deep learning library. The scenario generated using the proposed approach was finally applied to the virtual simulator, which was made by us, based on Unity for autonomous vehicle’s smart sensors and devices to train. Artificial intelligence objects such as people, animals, and cars exist in the virtual simulator and act based on artificial intelligence according to the input scenario. Based on the input scenario, human, animal, and vehicle agents are operated in front of an autonomous car. The autonomous vehicle’s virtual sensing device is trained by using RGB, depth, Lidar, and Radar data. Figure 6 shows the virtual simulator environment screenshot.

Studies analyzing driving videos via deep learning have been actively conducted using public driving datasets [21,22]. However, the public driving data have only single action labels. Accordingly, the experiment in this paper collected videos, including events that occurred on roads or streets, and labelled their ground truth. In total, 725 videos were collected and classified into 23 classes. Table 2 summarizes the event class types. The event classes have high-level event classes, including single actions of cars, animals, and people and the interactions among them. 

As shown in Table 3, nine object types were identified from the analysis on the objects included in each event. 

### 4.2. High-Level Event Area Extraction Results

**Figure 7:**
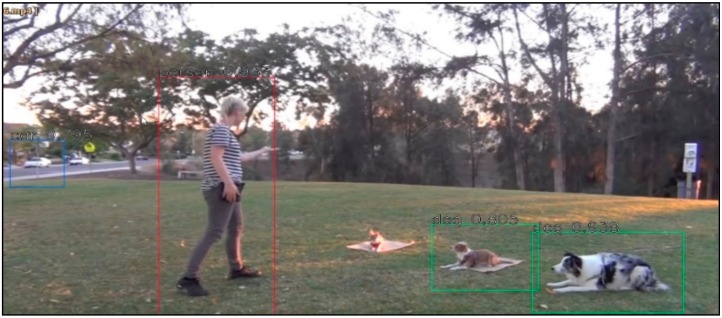
Faster-region based convolution neural network (RCNN) results.

**Figure 8:**
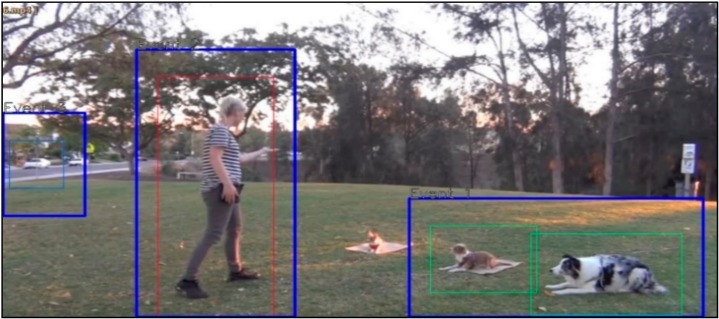
Faster-RCNN based event area integration algorithm results.

This subsection analyzes the Faster-RCNN-based event image extraction results. Although only the areas of each object are extracted, as shown in Figure 7, when extracting event areas only by using the existing Faster-RCNN, it is verified that the high-level event areas including objects that are correlated one another are extracted when the event area integration algorithm is applied as well, as shown in Figure 8.

### 4.3. LRCN-Based Event Classification Result

To analyze the extracted event images, the image analysis model was implemented based on the LRCN model combining CNN and LSTM. Next, an autonomous vehicle learned using the collected data and the accuracy of event classification was evaluated. Cross-validation, one of the methods to measure the effectiveness of classification performance in the field of computer vision recognition, was adopted to verify the learning model in this study. Cross-validation is a representative method to measure the accuracy by comparing the estimates with actual values when verification data is entered into the model after learning. Table 4 presents the confusion matrix with estimates and actual values. 

Accuracy indicates how close the measured values are to the true values. Equation (1) estimates the accuracy based on the confusion matrix in Table 4.

(1) $$\mathrm{Accuracy}\;=\;\frac{TP+TN}{TP+FN+FP+FN}.$$

**Figure 9:**
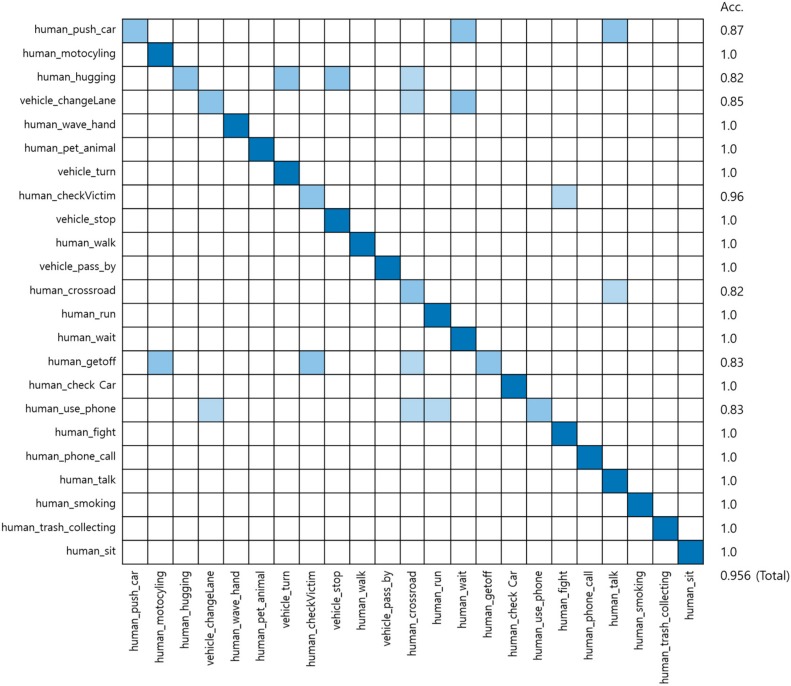
Confusion matrix of the LRCN result.

We applied Inception-v3 [23] which is a pre-trained model of CNN to LRCN. The training data was divided into 600 for training and 125 for testing. The input data size is 240 × 240 and the batch sizes are 34 for 200 epochs and two for 600 epochs. Figure 9 shows the confusion matrix of the result. 

The Table 5 presents the comparison results of LRCN and the proposed method. The proposed approach’s classification accuracy exceeds 96.5%.

### 4.4. Scenario Generation and Implementation Results 

Using the trained proposed model, we generated four scenarios as below. Table 6, Table 7, Table 8 and Table 9 show the input original video data which was taken in real world and the scenario generated by analyzing the driving video using deep learning and the results of implementing that scenario in the simulator. The implementation of this scenario verified that the objects detected from real driving data were analyzed per event unit and saved to the scenario file, and relevant multiple events were generated through artificial intelligence objects in the virtual simulator based on the scenario. 

## 5. Conclusions

This paper proposed an approach to automatically analyze real driving data by using the deep learning image analysis method without complicated scenario generation modeling and then generated a scenario for smart sensor and devices of an autonomous vehicle such as camera, Lidar, or Radar to train in virtual simulator, including multiple events based on the automatic analysis results. The approach proposed in this paper includes multiple events by extracting them from one driving image and enables high-level event analysis, including interactions among objects, not rather than analyzing only a single action of an object. 

The experiment achieved an accuracy of 95.6% by training the model using the dataset constructed in this paper and evaluating the event analysis model classified into 23 classes. Accordingly, it was verified that multiple high-level events could be acquired from a single video as compared to the existing deep-learning algorithm. Furthermore, it was observed that multiple events extracted were saved as one scenario and executed in a similar manner as the input driving data in the virtual self-driving simulator. 

Further studies must investigate extraction of dynamic events while tracking dynamically moving objects by analyzing all consecutive frames of a video when extracting the event areas. Moreover, further studies will attempt to determine the approach to enable analysis on a wide range of elements, including the movement direction and speed of an object, individual actions, weather, and road conditions included in the events as well as the event types. 

## Figures and Tables

**Table 1:** List of scenario elements.

Elements	Symbols	Description
Scenario	s = (e list)	One scenario includes multiple events
Event	e = (o list, event class)	An event includes the list of objects included in the event and the class of the event
Object	o = object	Objects including persons, animals, or cars
High-level event class	c = event class	Types of events occurring in the driving video

**Table 2:** List of event types.

High-Level Event Class	No. of Video Clips
human_push_car	36
human_motocyling	27
human_hugging	85
vehicle_changeLane	16
human_wave_hand	20
human_pet_animal	12
vehicle_turn	24
human_checkVictim	22
vehicle_stop	50
human_walk	53
vehicle_pass_by	70
human_crossroad	39
human_run	13
human_wait	81
human_getoff	15
human_check Car	5
human_use_phone	7
human_fight	10
human_phone_call	27
human_talk	12
human_smoking	53
human_trash_collecting	24
human_sit	24
23	725

**Table 3:** List of object types.

Object Types (Total Nine Types)
Person, car, bike, motorbike, bus, truck, bird, cat, dog

**Table 4:** Confusion of estimates and actual values.

Confusion Matrix		Actual Values	
		Positive	Negative
**Estimates**	**Positive**	True Positive (TP)	False Positive (FP)
	**Negative**	False Negative (FN)	True Negative (TN)

**Table 5:** Comparison results of classification models.

	LRCN [19]	LRCN + Full Area	LRCN (Inception-v3) + Full Area (Proposed Approach)
Classification Accuracy	78.2	80.5	95.6

**Table 6:** Scenario Generation Result #1.

Input Data
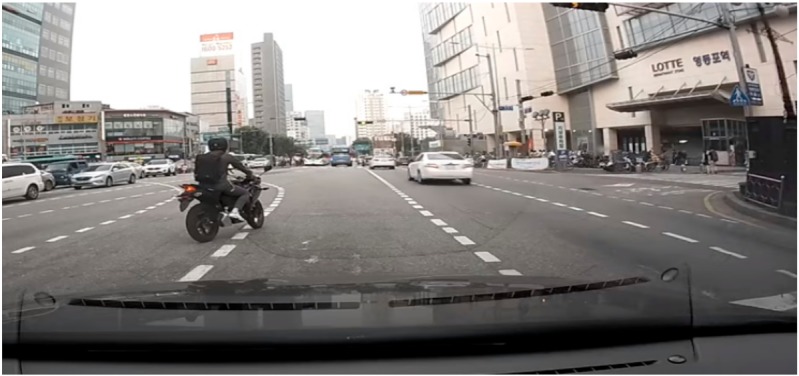
**Output data (= scenario)**
s1 = {e1(human_motocyling), e2(vehicle_changeLane)}
**Final result in simulator**
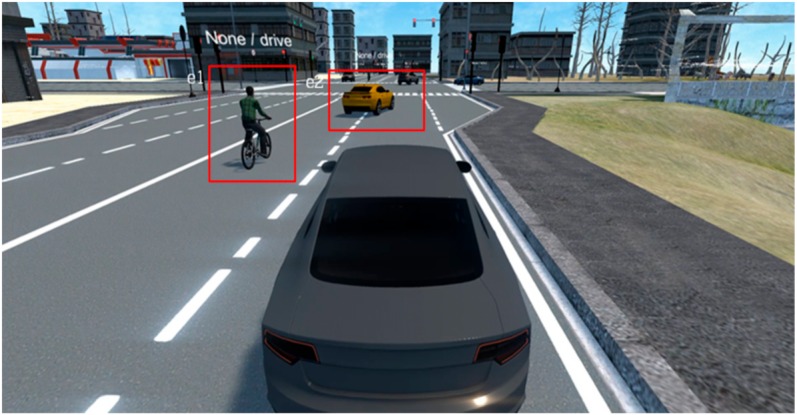

**Table 7:** Scenario Generation Result #2.

Input Data
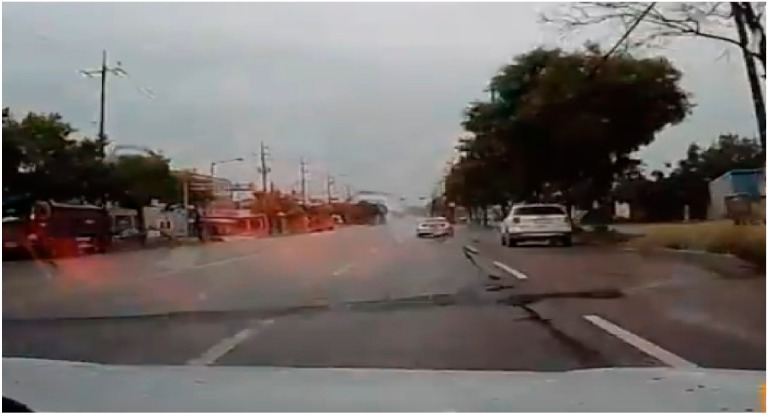
**Output data (= scenario)**
s2 = {e1(vehicle_turn), e2(vehicle_stop)}
**Final result in simulator**
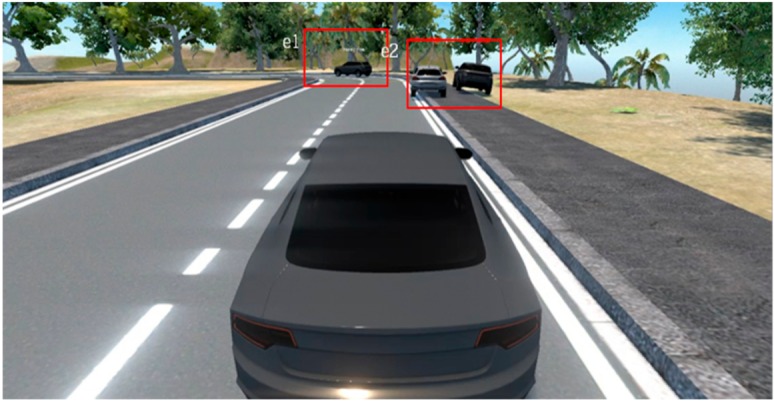

**Table 8:** Scenario Generation Result #3.

Input Data
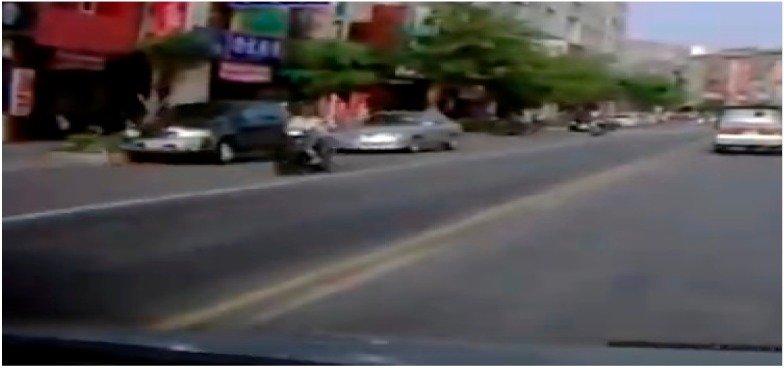
**Output data (= scenario)**
s3 = {e1(vehicle_stop), e2(human_motocyling e3(vehicle_stop)}
**Final result in simulator**
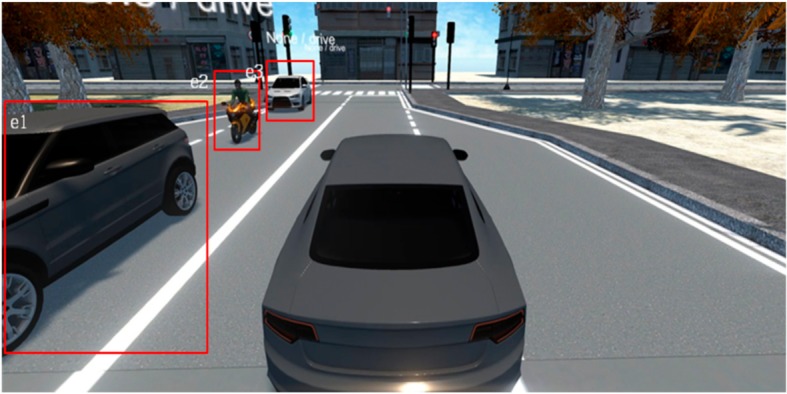

**Table 9:** Scenario Generation Result #4.

Input data
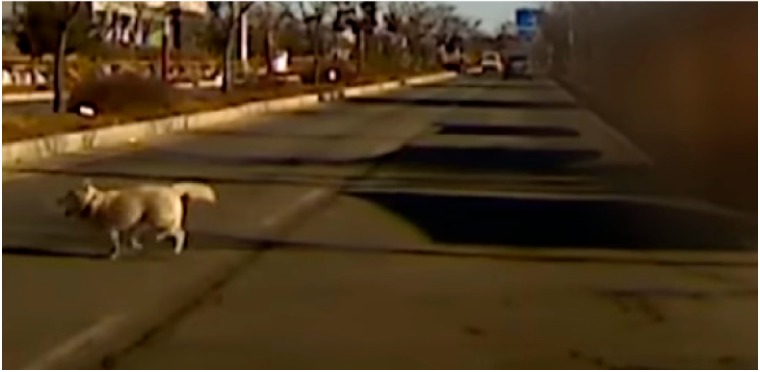
**Output data (= scenario)**
s4 = {e1(human_pet_animal), e2(vehicle_stop)}
**Final result in simulator**